# The disturbance of ABC transporters in patients with dengue fever: integration of metabolomics and transcriptomics

**DOI:** 10.3389/fcimb.2025.1749051

**Published:** 2026-02-11

**Authors:** Chengxin Liu, Bei Ye, Kai Wang, Jiafang Chen, Huiting Huang, Yong Jiang, Geng Li, Shaofeng Zhan

**Affiliations:** 1 The First Affiliated Hospital, Guangzhou University of Chinese Medicine, Guangzhou, China; 2 The First Clinical Medical School, Guangzhou University of Chinese Medicine, Guangzhou, China; 3 Shenzhen Hospital of Integrated Traditional Chinese and Western Medicine, Shenzhen, China; 4 Laboratory Animal Center, Guangzhou University of Chinese Medicine, Guangzhou, China

**Keywords:** ABC transporters, dengue fever, metabolic biomarkers, transcriptomics, untargeted metabolomics

## Abstract

**Background:**

DENV virus (DENV) infection can cause various symptoms and organ damage, even severe dengue fever. However, the underlying host response products and interfering metabolic pathways and mechanisms of DENV infection remain unclear. In this study, we characterized the metabolites and metabolic pathway changes during DENV infection using liquid chromatography- (LC-MS) and gas chromatography-mass spectrometry (GC-MS). And identify the hub differentially expressed targets associated with major metabolism pathways combining transcriptomics.

**Methods:**

Plasma from adult patients infected with DENV infection was characterized by untargeted metabolomics using LC-MS and GC-MS. Potential diagnostic biomarkers for dengue fever were indicated using ROC curve analysis. KEGG and GSEA functional enrichment analysis was the strategy to determine the mechanisms of key metabolic pathways in dengue fever. Potential targets were identified by combining transcriptomic in Gene Expression Omnibus (GEO) datasets, and gene databases from GeneCards and the Comparative Toxicogenomics Database (CTD).

**Results:**

A total of 41 dengue patients and 23 healthy volunteers were recruited for the study. 61 up-regulated and 136 down-regulated metabolites were identified via untargeted metabolomics. The top10 up-regulated metabolites with high AUC values included trans-cinnamic acid, L-Acetylcarnitine, SM(d17:1/17:0), 1,2,4,5-cyclohexanetetrol, 5-(hydroxymethyl) pyrrolidin-2-one, 1,2,3,4-tetrahydro-6-propanoylpyridine, 2-C-methyl-D-erythritol-4-phosphate, Physalolactone, S-Japonin, and 9-tridecynoic acid, and they were supposed to be the potential diagnostic biomarkers for dengue fever. The disturbance of ATP-binding cassette (ABC) transporters, protein digestion and absorption, aminoacyl-tRNA biosynthesis, mineral absorption, and D-amino acid metabolism were enriched in the metabolic pathways. ABCC5, ABCB1, and ABCG5 were identified as hub differentially expressed targets through transcriptome profiling and protein-protein interaction networks.

**Conclusions:**

The current study revealed a shift in metabolite profiles and disturbance in ABC transporters in dengue fever, which can be used for further functional verification.

## 1 Introduction

Dengue fever is an acute infectious disease caused by dengue virus (DENV), transmitted by mosquitoes of the genus *Aedes* (Wilder-Smith et al., 2019). With a significant increase in transmission over the past few decades, dengue fever has become the most rapidly spreading viral disease globally. The World Health Organization (WHO) reports that by 2023, the number of reported dengue cases in more than 80 countries/areas will be close to its highest level ever, with more than 5 million cases and more than 5,000 dengue-related deaths (https://www.who.int/emergencies/disease-outbreak-news/item/2023-DON498, Last update time 21 December 2023). However, these figures may underestimate the true severity, as most primary infections are asymptomatic, and mandatory reporting is not required in many countries. Previous modeling estimate indicates that 390 million people are infected with DENV annually, 96 million of whom are clinically active, with 70% presenting in Asia (Bhatt et al., 2013). Various factors, such as the rise in viral diversity (Salje et al., 2017) and the expanding habitats of mosquito vectors *Aedes* (Kraemer et al., 2019), are expected to increase the prevalence of dengue in the coming decades, posing a major threat to global public health and a huge economic burden to the world.

The clinical symptoms of DENV infection range from asymptomatic infection to a potentially fatal dengue shock syndrome. The most typical clinical symptoms of dengue fever are pyrexia and body aches, which may last 5–7 days and are accompanied by nausea, vomiting, rash, and exhaustion for several days (Rigau-Perez et al., 1998). Most patients will gradually improve, but some will develop severe dengue, usually manifested by severe plasma leakage, severe bleeding, or severe organ failure. There are no specific treatments other than supportive care, and symptomatic treatment (including antipyretic and analgesic) has become the main therapeutic aim of dengue fever. There is no doubt that preventing DENV infection is crucial to reducing the incidence of dengue fever, and therefore, the development of a dengue vaccine has become a major research priority (Kariyawasam et al., 2023).

It is crucial to study the host response products, and metabolomics provides a means to investigate the wide range of changes during human infection by detecting aberrant levels of metabolites (Byers et al., 2019). A previous study uses metabolomics to find decreased serum levels of serotonin in dengue patients and predicts that it can be used as a marker for early diagnosis of dengue hemorrhagic fever (DHF) (Cui et al., 2016). The substrates and product changes analyzed by metabolomics can provide a phenotypic readout for clinical diagnosis, identification of therapeutic targets for diseases, and in-depth studies of fundamental biological processes (Patti et al., 2012). Metabolomics measures chemical phenotypes downstream of genomic, transcriptomic, and proteomic variability, offering a highly integrated profile of biological status. There is increasing research using metabolomics to reveal pathophysiologic mechanisms of disease (Newgard, 2017).

Transcriptomic strategies are widely used in biomedical research fields to reveal gene expression changes for assessing health status, disease recurrence, or mutational status (Lowe et al., 2017). Transcriptomics can be used to study the connection between gene expression and phenotypic heterogeneity after DENV infection. In our earlier study, a mouse model of DENV infection was established, and transcriptomics was used to explore the mechanisms of DENV-induced hepatic injury (Zheng et al., 2022).

No studies are comparing the differences between dengue patients and healthy volunteers by integrating metabolomics and transcriptomics. In this study, we characterized the metabolome changes during DENV infection using LC-MS and GC-MS. We also obtained the gene expression differences between dengue patients and healthy people through the transcriptomic method. Potential biomarkers of dengue were identified by ROC profile analysis. These findings may have important clinical significance for the early detection of dengue fever and the development of treatment strategies against DENV infections.

## 2 Materials and methods

### 2.1 Patient source and diagnostic criteria

A total of 41 dengue patients and 23 healthy volunteers were recruited at the First Affiliated Hospital of Guangzhou University of Chinese Medicine in 2023. According to the Guidelines for the diagnosis and treatment of dengue in China (2018) published by the Chinese Society of Infectious Diseases, Chinese Medical Association, patients were diagnosed with dengue fever if they met the following criteria: 1) epidemiological history of recent travel to dengue endemic areas; 2) fever, weak, muscle or joint pain, rash, and even bleeding tendency; 3) decreased peripheral white blood cells or platelets; 4) DENV IgM, NS1 antigenemia, or DENV nucleic acid positivity. Demographic and clinical data of dengue patients in the study were obtained from electronic medical records and case report forms.

### 2.2 Sample collected and stored

The blood samples were collected in the morning on an empty stomach using standard venipuncture procedures. To isolate plasma, heparin tubes were used for anticoagulants. Plasma samples were separated by centrifuging at 3,000 g for 10 min, collected into new centrifuge tubes, transferred to a Thermo Scientific 70U Series freezer, and stored at -80°C. This study was approved by the Ethical Committee of First Affiliated Hospital of Guangzhou University of Chinese Medicine (NO. K-2022-066). All examinees in both dengue fever and healthy groups voluntarily joined this study with informed consent signed. Metabolomic data analysis was entrusted to be performed by Shanghai Luming Biological Technology Co., LTD (Shanghai, China).

### 2.3 Sample preparation

150 μL of the sample and 600 μL protein precipitant (a mixture of L-2-chlorophenylalanine, succinic acid-d4, L-Valine-d8, and bile acid-D4 dissolved in methanol-acetonitrile as internal standard, with a volume ratio of the two solvents of 2:1, 4 μg/mL) were mixed and vortexed for 10 s. Subsequently, the whole samples were extracted by ultrasonic for 10 min in an ice-water bath, and stored at -40°C overnight. The next day, the samples were centrifuged at 4°C (12000 rpm) for 10 min. The supernatant (150 µL) was removed from each tube, filtered through a 0.22 µm microfilter, and transferred to an LC vial. Samples were stored at -80°C until LC-MS analysis.

150 μL of supernatant was transferred to a glass sampling vial and dried under vacuum at room temperature using a centrifugal concentration dryer. Then 80 μL of methoxylamine hydrochloride in pyridine (15 mg/mL) was added and oxidized for 60 min at 37°C in a steam bath shaker. After removing the sample, 50 μL of BSTFA and 20 μL n-hexane were added to the mixture, with 10 μL of the internal standard mixtures, which was vortexed vigorously and derivatized for 60 min at 70°C. The mixture of internal standards contained the following reagents, indicated by Cat Number: G162300, N-9M-AU4-B, N-10M-A18-D, N-12M-AU15-D, N-14M-A24-E, G161798, N-18M-O9-C, N-20M-J27-E, N-22M-JY30-E, and N-24M-S6-A. Finally, the samples were left at room temperature for 30 min followed by GC-MS analysis.

The quality control (QC) sample was prepared by mixing equal volumes of extracts from all samples to evaluate the sample preparation, derivatization, sample loading, and the stability of the mass spectrometry system during sample testing.

### 2.4 LC-MS-based untargeted metabolomics analysis

An Ultra High-Performance Liquid Tandem High-Resolution Mass Spectrometer (UHPLC-HRMS) (Waters ACQUITY UPLC I-Class plus/Thermo QE) was used to analyze the metabolic profiling in both positive and negative ion modes. An ACQUITY UPLC HSS T3 Chromatography Column (1.8 μm, 100 mm×2.1 mm) was used in both positive and negative modes. The binary gradient elution system consisting of (A) water (containing 0.1% formic acid) and (B) acetonitrile was used to achieve the separation: 0 min, 5% B; 2 min at 5%B; 4 min at 30%B; 8 min at 50%B; 10 min at 80%B; 14 min at 100%B; 15 min at 100%B; 15.1 min at 5%B and 16 min, 5%B. The flow rate was 0.35 mL/min and the column temperature was 45°C. All samples were stored at 10°C during analysis, and the injection volume was 3 μL.

The mass spectrometer was operated as follows: Spray voltage, 3800 V (+) and 3000 V (−); Capillary temperature, 320°C; Aux gas heater temperature, 350°C; Sheath gas flow rate, 35 arbitrary units; Auxiliary gas flow rate, 8 arbitrary units; S-lens RF level, 50. The mass range was from m/z 70 to 1,050. The resolution was set at 70,000 for the full MS scans and 17500 for MS/MS. The NCE/stepped NCE was set at 10, 20 and 40 eV.

### 2.5 GC–MS-based untargeted metabolomics analysis

The derivatized samples were analyzed on an Agilent 7890B-5977A gas chromatography system. Derivatives were separated using a DB-5MS fused silica capillary column (30 m × 0.25 mm × 0.25 μm, Agilent J & W Scientific, Folsom, CA, USA). The carrier gas was helium (> 99.999%), and the flow rate was 1.0 mL/min. The syringe temperature was maintained at 260 °C. A splitless mode was used with an injection volume of 1 μL. The oven was held at an initial temperature of 60°C for 0.5 min, then increased to 125°C at a rate of 8°C/min, 210°C at a rate of 8°C/min, 270°C at a rate of 15°C/min, 305°C at a rate of 20°C/min, and finally held at 305°C for 5 min. The temperature of the ion source (electron impact, EI) and the MS quadrupole was set to 230°C and 150°C, respectively. The collision energy was 70 eV, and the mass spectrometric data was acquired in a full-scan mode (m/z 50-500).

### 2.6 Data preprocessing and normalization

Raw LC-MS data were baseline filtered, peaks identified, integrated, retention time corrected, peak aligned and normalized using Progenesis QI v3.0 software (Nonlinear, Dynamics, Newcastle, UK). The main parameters of precursor tolerance 5 ppm and product tolerance 10 ppm were based on The Human Metabolome Database (HMDB) and Lipidmaps (v2.3), while 10 ppm precursor tolerance and 20 ppm product tolerance were based on LuMet-Animal v3.0 and METLIN databases. The compounds were identified based on the above databases according to several dimensions of retention time, the precise mass-to-charge ratio (M/z), secondary fragments, and isotopic distribution. The extracted data were then further processed by removing all peaks in the group with more than 50% missing values (ion intensity = 0), replacing the zero values with half of the minimum value and screening compounds based on qualitative results. Compounds scoring less than 36 points out of 80 were also considered inaccurate and removed.

The raw D-format GC/MS data obtained were converted to abf format for quick data retrieval using Analysis Base File Converter software. The data were then imported into MS-DIAL v4.24 software for peak detection, peak identification, MS2Dec deconvolution, characterization, peak alignment, wave filtering, and missing value interpolation, and finally the data matrix. Metabolite characterization was based on the LuMet-GC 5.0 database and NIST Chemistry Web Book. All internal standard peaks and pseudo-positive peaks were then excluded and further processed to exclude all peaks in the group with more than 50% missing values (ionic intensity = 0) and replace the zero value with half of the minimum value. The signal intensities of all peaks in each sample were split and normalized based on internal standards with a filtered RSD greater than 0.1. Redundancy removal and peak merging were then performed, and compounds with scores below 70 out of 100 were also considered inaccurate and removed. In each sample, all peak signal intensities were segmented and normalized according to the internal standards with RSD greater than 0.1 after screening. After the data was normalized, redundancy removal and peak merging were conducted to obtain the data matrix. Compounds scoring less than 70 points out of 100 were also considered inaccurate and removed.

The positive and negative ion data from the LC-MS/MS and the data obtained from the GC-MS were combined into a data matrix that contained all the information extracted from the raw data and could be used for subsequent analysis.

### 2.7 Statistical analysis of metabolomics

Principle Component Analysis (PCA) was used to observe the overall distribution of the samples and the stability of the whole analytical process. To distinguish the different metabolites between groups, orthogonal partial least squares discriminant analysis (OPLS-DA) was used. To prevent overfitting, the quality of the model was evaluated using 7-fold cross-validation and 200 Response Permutation Testings (RPTs). The Variable important in projection (VIP) values from the OPLS-DA model were used to rank the contribution of each variable to group discrimination. Additionally, a *two-tailed Student’s T-test* was used to verify the significance of the differences in metabolites between groups. We selected differential metabolites based on VIP values greater than 1.0 and *P*-values less than 0.05.

### 2.8 Transcriptome data of dengue fever and targets of ABC transporters

The RNA-seq data were downloaded from Gene Expression Omnibus (GEO, https://www.ncbi.nlm.nih.gov/geo/) with GEO series accession numbers GSE51808, GSE96656, and GSE206829, the differentially expressed genes (DEGs) were obtained using the R programming language. The populations for these 3 datasets included dengue patients and healthy volunteers (Rao et al., [[NoYear]]; Kwissa et al., 2014; Popper et al., 2018). Further, targets in the ABC transporters pathway were also obtained from GeneCards (https://www.genecards.org/) and the Comparative Toxicogenomics Database (CTD, https://ctdbase.org/). The intersection of dengue fever and ABC transporters was obtained via Venn to focus on the potential core targets. The protein-protein interaction (PPI) network was constructed by STRING 12.0 (https://string-db.org/). CytoHubba, a Cytoscape plugin (version 3.10.1), was used to find the hub genes. A further whole peripheral blood was collected in part of the subjects. The TRIzol reagent (Invitrogen, CA, USA) was used to extract the total RNA. Then cDNA was generated from the extracted RNA using the HiScript II Reverse Transcriptase. The primer Ct value was detected by real-time fluorescence quantitative PCR(RT-qPCR) three times. The non-parameter tests were performed to calculate the difference in expressed genes of sequencing data and RT-qPCR analyses.

## 3 Results

### 3.1 Demographic characteristics

The population characteristics of the dengue fever patients and healthy volunteers were presented in **Table 1**. There were no significant differences in the sex ratio between the two groups (*P* < 0.05). There was a statistical difference in age between the two groups, with the patient group being slightly older than the volunteer group. Nonetheless, it is unlikely that subtle age differences would cause any bias in the results of metabolomics studies. The patients were all seen within 1 to 5 days of the acute disorder. Among them, 7 dengue patients were infected with DENV-1 and 34 with the DENV-2 serotype.

**Table 1:** Population characteristics of the patients and healthy volunteers.

	Dengue fever/n=41	Healthy volunteers/n=23	χ^2^/Z	*P*
Gender (male/female)	18/23	11/12	0.092	0.762
Age (years)	40.78 ± 11.195	33.30 ± 11.570	-2.591	0.010
Course of disease (days)	2.59 ± 1.284	NA	/	/
Serotypes (I/II)	7/34	NA	/	/

### 3.2 Modeling and score plots of multivariate statistical analysis

**Figure 1:**
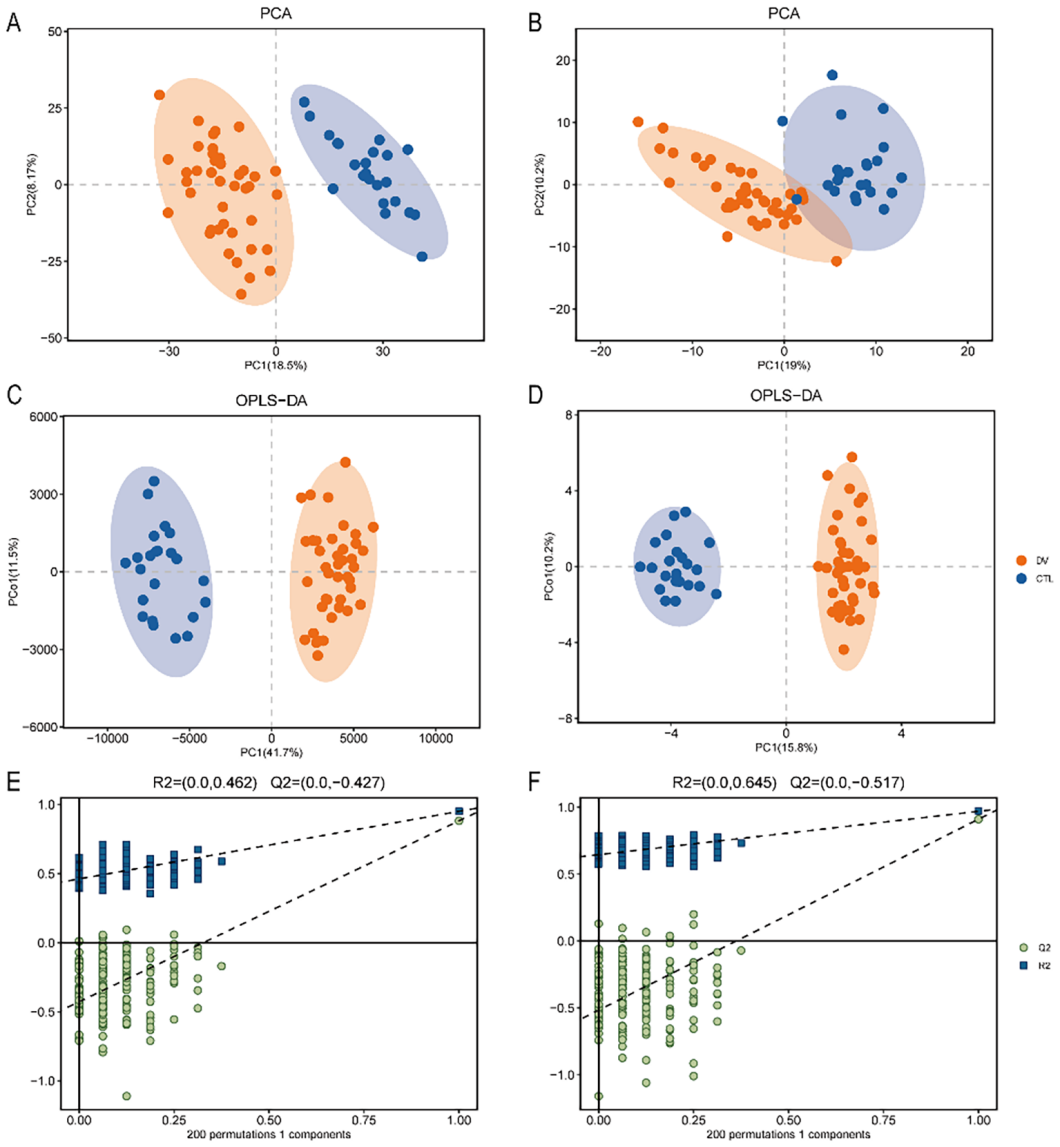
Altered metabolic profiles in patients with dengue fever compared to healthy volunteers. **(A, B)** Score plot of PCA based on LC-MS-and GC–MS untargeted metabolomics analysis. **(C, D)** The differences in metabolic phenotypes in plasma between dengue fever patients and healthy volunteers were analyzed by OPLS-DA, a supervised multivariate data analysis method, using LC-MS-based and GC-MS-based. **(E, F)** Statistical validation of the OPLS-DA model by permutation testing with 200 iterations. Of them, **Figure 2A, C, E** were LC-MS based, and **Figures 1B, D, F** were GC-MS based.

PCA and OPLS-DA were used to analyze the LC/MS and GC/MS data obtained from two sets of plasma metabolome sequencing. On the PCA score plot, samples in the same group were clustered closer to each other, while the coordinate points of different samples with significant differences were relatively far away. The OPLS-DA is corrected from PLS-DA (Partial least squares discriminant analysis) by filtering out noise not related to categorical information. In **Figures 1A–D**, PCA and OPLS-DA analyses clearly distinguished the dengue fever group (orange dots) and the healthy group (blue dots), indicating significant differences in plasma metabolites between the two groups.

Splot diagrams were drawn to represent the eigenvalues and correlations of the metabolites, with metabolites closer to the upper right and lower left corners indicating more significant differences. Both LC/MS (**Figure 1E**, R2 = 0.462, Q2 = −0.427< 0) and GC/MS (**Figure 1F**, R2 = 0.645, Q2 = −0.517< 0) obtained good predictions and avoided overfitting.

### 3.3 Identification of differentially expressed metabolites in plasma

**Figure 2:**
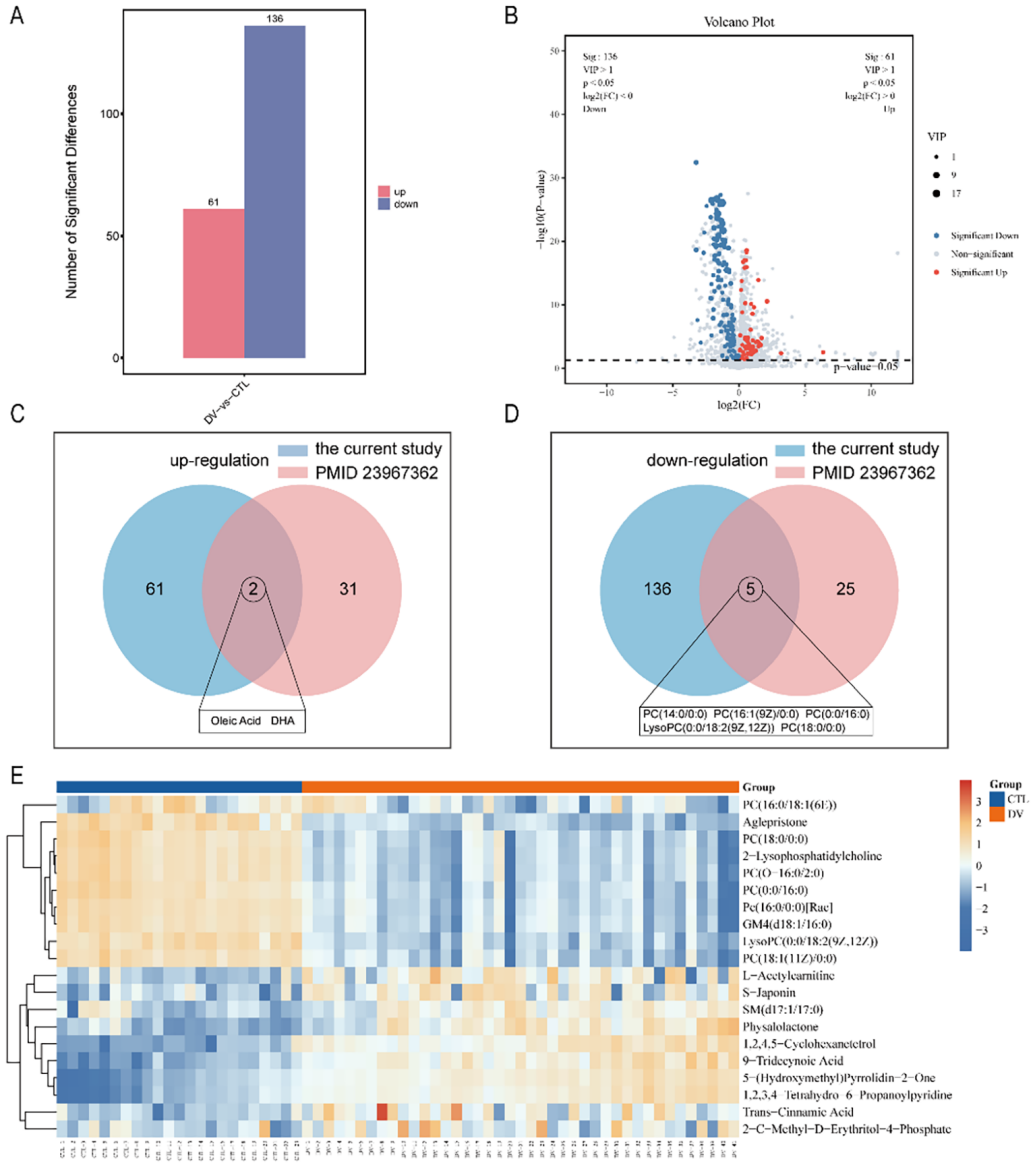
the composition differences in metabolite, the criteria used for screening differentially expressed metabolites was VIP>1 based on OPLS-DA model analysis and significant inter-group differences based on the t-test (P<0.05). The differential metabolites were exhibited using a column graph **(A)**, volcano Plot **(B)**, and heat map **(E)**. Compared with previous studies (PMID 23967362), the shared differences in metabolism in dengue patients included 2 up- and 5 down-regulation **(C, D)**.

A total of 2,759 metabolites were identified using an integrated method of LC-MS and GC-MS-based untargeted metabolomics. The criteria for screening differentially expressed metabolites was VIP>1 based on OPLS-DA model analysis and significant inter-group differences based on the t-test (P<0.05). There were 197 metabolites significantly changed in the dengue group compared to the healthy group, of which 61 were up-regulated and 136 were down-regulated (**Figure 2A**). The volcano plots showing the differential metabolites were shown in **Figure 2B**.

To better point out the relationship between the samples and the up-and down-regulation differences in metabolite expression between the two groups, heatmap analysis and plotting were performed for VIP Top10 up- and down-regulated metabolites. As shown in **Figure 2E**, compared to healthy control volunteers, the metabolites in plasma samples significantly altered in dengue fever patients, as indicated by increased levels of trans-cinnamic acid, L-acetylcarnitine, SM(d17:1/17:0), 1,2,4,5-Cyclohexanetetrol, 5-(hydroxymethyl)pyrrolidin-2-One, 1,2,3,4-tetrahydro-6-propanoylpyridine, 2-C-methyl-D-erythritol-4-phosphate, physalolactone, S-japonin, and 9-tridecynoic acid, as well as decreased levels of Pc(16:0/0:0)[Rac], 2-lysophosphatidylcholine, lysoPC (0:0/18:2(9Z,12Z)), PC (0:0/16:0), GM4 (d18:1/16:0), PC (18:0/0:0), PC (16:0/18:1(6E)), PC (18:1(11Z)/0:0), aglepristone, and PC (O-16:0/2:0). Furthermore, 2 up-regulated and 5 down-regulated shared metabolites were identified compared with previous related studies (**Figures 2C, D**).

**Figure 3:**
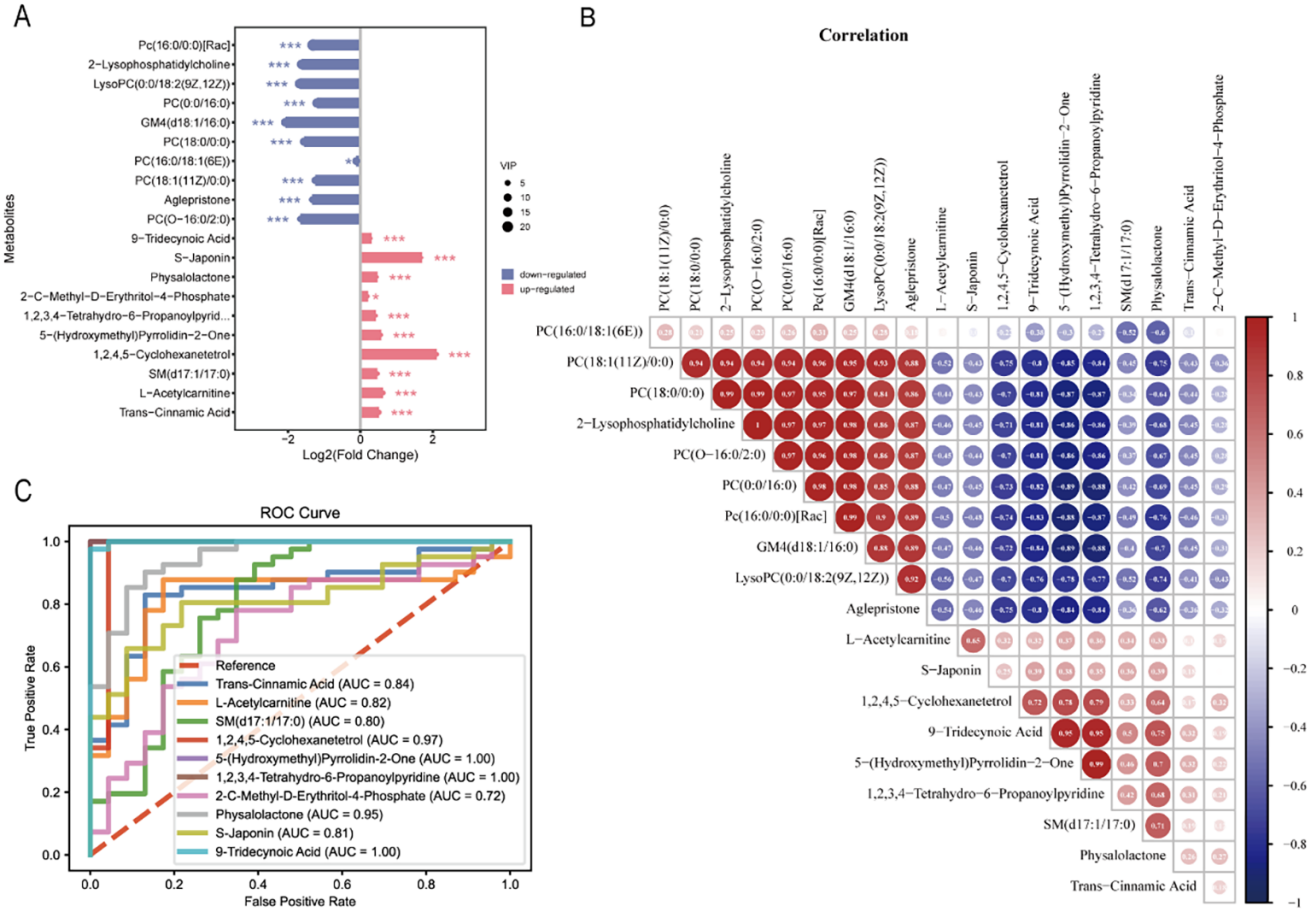
lollipop plot and correlation plot at the VIP Top10 up- and down-regulated metabolites. **(A)** lollipop plot shows the significant changes in metabolite levels. Red for upregulation and blue for downregulation. The abscissa represents |Log FC|, the circle size indicates VIP values, * indicates P< 0.05, *** indicates P< 0.001. **(B)** the correlation plot was constructed in the Top10 up- and down-regulated metabolites, with red circles representing positive correlations and blue circles representing negative correlations, the size and darkness in the circles indicate the strength of the correlation. **(C)** the ROC curves for the most significantly up-varied differential metabolites. In the ROC curve, the true positive rate (TPR, indicates sensitivity) is plotted on the ordinate, and the false positive rate (FPR, indicates 1-specificity) is plotted on the abscissa. The closer the AUC is to 1, the better the model.

**Figure 3A** showed the distribution of the top 10 up- and down-regulated differential metabolites between the two groups. In addition, the top 10 up- and down-regulated metabolites were also correlated, and there was a negative correlation between the up- and down-regulated metabolites (**Figure 3B**). The power calculations of top 10 up-regulated metabolites were listed (**Table 2**).

**Table 2:** The power calculations of top 10 up-regulated metabolites.

Metabolites	Formula	VIP	FC	Regulation	p-value
Trans-Cinnamic Acid	C9H8O2	3.860	1.432	Up	0.00045
L-Acetylcarnitine	C9H17NO4	3.647	1.547	Up	2.1E-05
SM(d17:1/17:0)	C39H79N2O6P	3.252	1.384	Up	2.1E-05
1,2,4,5-Cyclohexanetetrol	C6H12O4	3.041	4.326	Up	2.7E-11
5-(Hydroxymethyl)Pyrrolidin-2-One	C5H9NO2	2.506	1.491	Up	2.6E-19
1,2,3,4-Tetrahydro-6-Propanoylpyridine	C8H13NO	2.475	1.349	Up	9.9E-18
2-C-Methyl-D-Erythritol-4-Phosphate	C5H13O7P	2.330	1.159	Up	0.01473
Physalolactone	C28H39ClO8	2.040	1.389	Up	5.4E-11
S-Japonin	C19H28O3S	1.996	3.265	Up	1.7E-05
9-Tridecynoic Acid	C13H22O2	1.988	1.239	Up	1.7E-17
Pc(16:0/0:0)[Rac]	C24H50NO7P	23.573	0.404	Down	8.6E-27
2-Lysophosphatidylcholine	C26H54NO7P	15.548	0.324	Down	4.3E-25
LysoPC(0:0/18:2(9Z,12Z))	C26H50NO7P	15.126	0.311	Down	2E-27
PC(0:0/16:0)	C24H50NO7P	15.013	0.435	Down	2E-22
GM4(d18:1/16:0)	C51H94N2O16	12.239	0.237	Down	3.3E-27
PC(18:0/0:0)	C26H54NO7P	10.311	0.339	Down	1.4E-21
PC(16:0/18:1(6E))	C42H82NO8P	9.248	0.921	Down	0.01228
PC(18:1(11Z)/0:0)	C26H52NO7P	8.713	0.422	Down	1.5E-23
Aglepristone	C29H37NO2	6.476	0.397	Down	5.5E-24
PC(O-16:0/2:0)	C26H54NO7P	6.346	0.316	Down	2.4E-24

Receiver operating characteristic curves (ROCs) were constructed using the top 10 up-regulated metabolites of VIP to differentiate dengue from healthy controls. The AUC values of each metabolite were 0.843, 0.824, 0.800, 0.971, 1.000, 1.000, 0.721, 0.948, 0.812 and 0.999 respectively (**Figure 3C**).

### 3.4 Metabolic pathway analysis

**Figure 4:**
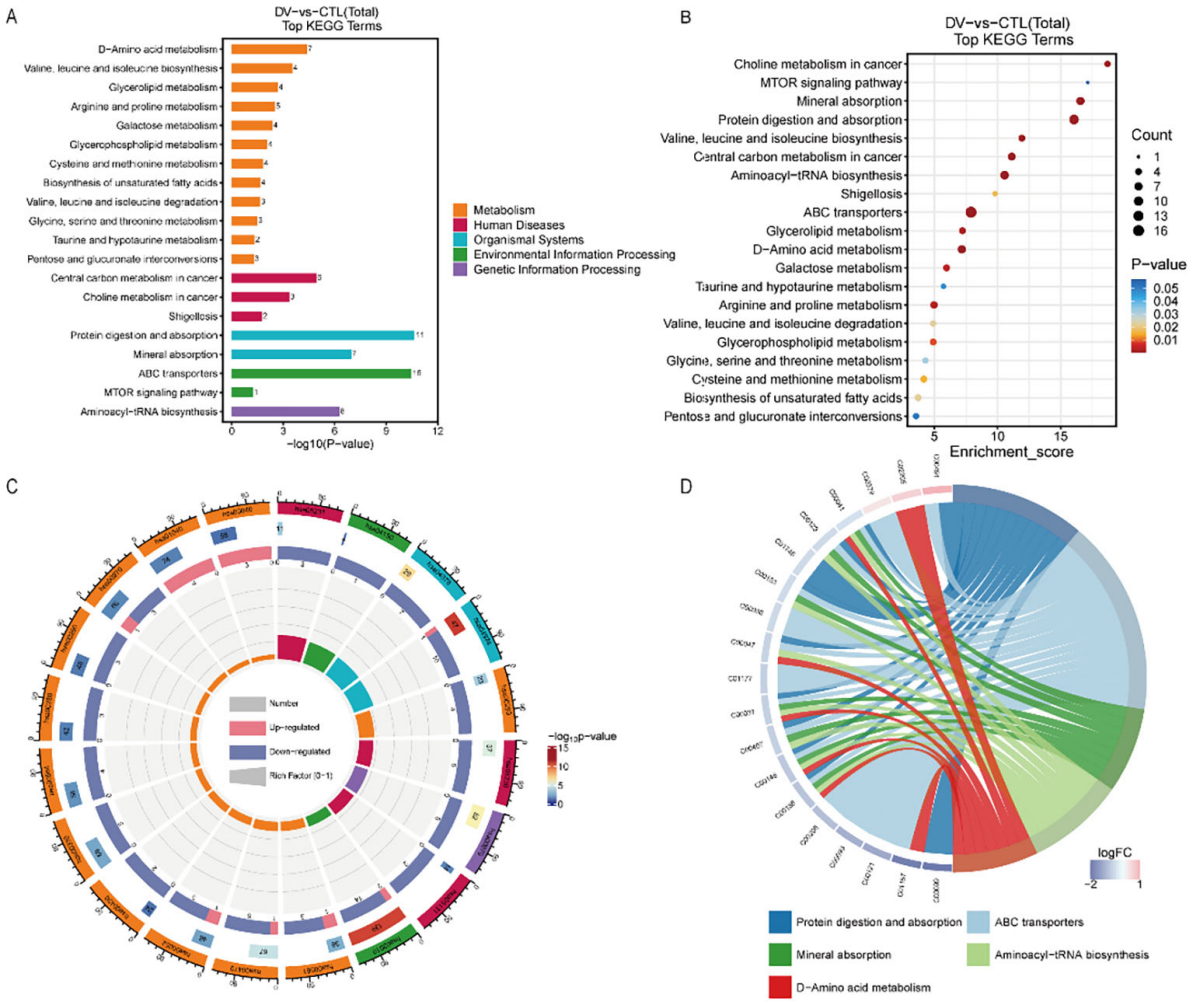
Metabolic pathway analysis based on the differentiated plasma metabolites. ABC transporters, Protein digestion and absorption, aminoacyl-tRNA biosynthesis, Mineral absorption, and D-Amino acid metabolism, were matched to the higher number of metabolites, and simultaneously significantly high enriched (P <0.05). **(A)** bar chart in Top20 KEGG Terms. **(B)** bubble chart in Top20 KEGG Terms. **(C)** doughnut in Top20 KEGG Terms. A total of 4 circles from outside to inside. The color in the first circle represents different KEGG pathway categories The number in the second circle represents PopHits, which means the number of all metabolites annotated to this pathway, and the depth of color from blue to red represents low to high expression, respectively. In the third circle, the red bar represents up-regulated metabolites, while the blue bar represents down-regulated metabolites. And in the last circle means the value of RichFactor. **(D)** chord diagram in Top5 KEGG Terms. The left side represents metabolites the right stands for the metabolic pathway, and the line connecting the two sides represents association enrichment.

Metabolites in plasma from both groups were mapped to metabolic pathways using the Kyoto Encyclopedia of the Genome (KEGG). ABC transporters, as well as Protein digestion and absorption, aminoacyl-tRNA biosynthesis, Mineral absorption, and D-Amino acid metabolism, were matched with a higher number of metabolites that were significantly enriched at the same time (*P* < 0.05). The top 20 significantly enriched metabolite pathways were shown in **Figures 4A–C**. In addition, the mainly perturbed metabolic pathways along with the metabolite data were shown in **Table 3**, **Figure 4D**.

**Table 3:** KEGG metabolic pathway analysis.

KEGG id	Term	List Hits	Pop Hits	q-value	Enrichment score
hsa02010	ABC transporters	16	139	1.30E-09	7.895
hsa04974	Protein digestion and absorption	11	47	1.30E-09	16.053
hsa00970	Aminoacyl-tRNA biosynthesis	8	52	9.76E-06	10.552
hsa04978	Mineral absorption	7	29	2.73E-06	16.556
hsa00470	D-Amino acid metabolism	7	67	0.000498	7.166

**Figure 5:**
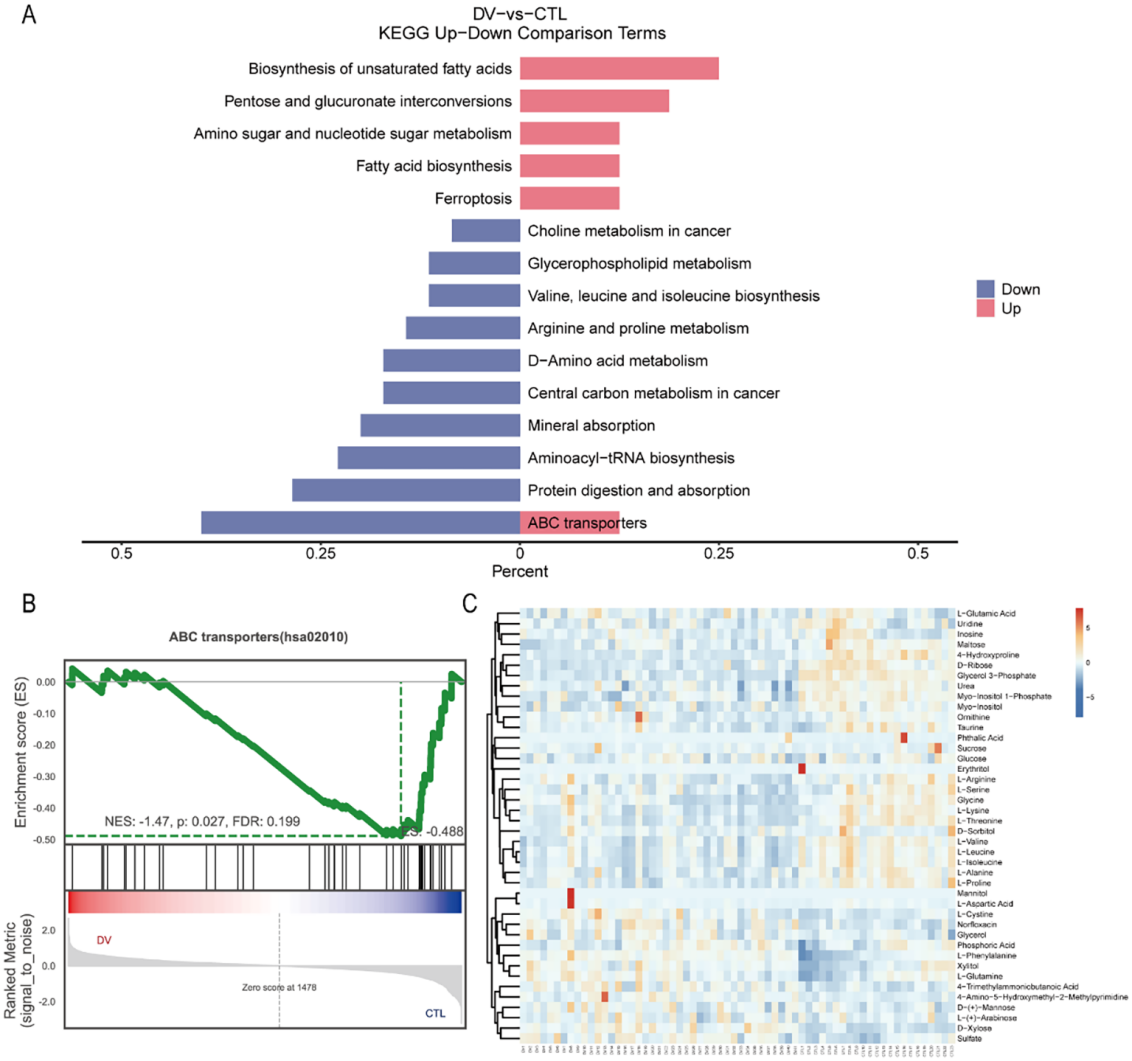
The disturbance in ABC transporters was identified as a characteristic change in the DENV-infected. **(A)** The pathway enrichment analysis for the up and down-regulated metabolites separately. For both bars, red and blue represent significant enrichment by the up-regulated and down-regulated metabolites, respectively. **(B)** ABC transporters-pathway suppression was confirmed through GSEA (NES= -1.47< -1, P-value= 0.027<0.05, FDR = 0.199<0.25). **(C)** heatmap showing GSEA metabolites expression. Relative expression is indicated by color, red indicates high expression, while blue indicates low expression.

**Figure 6:**
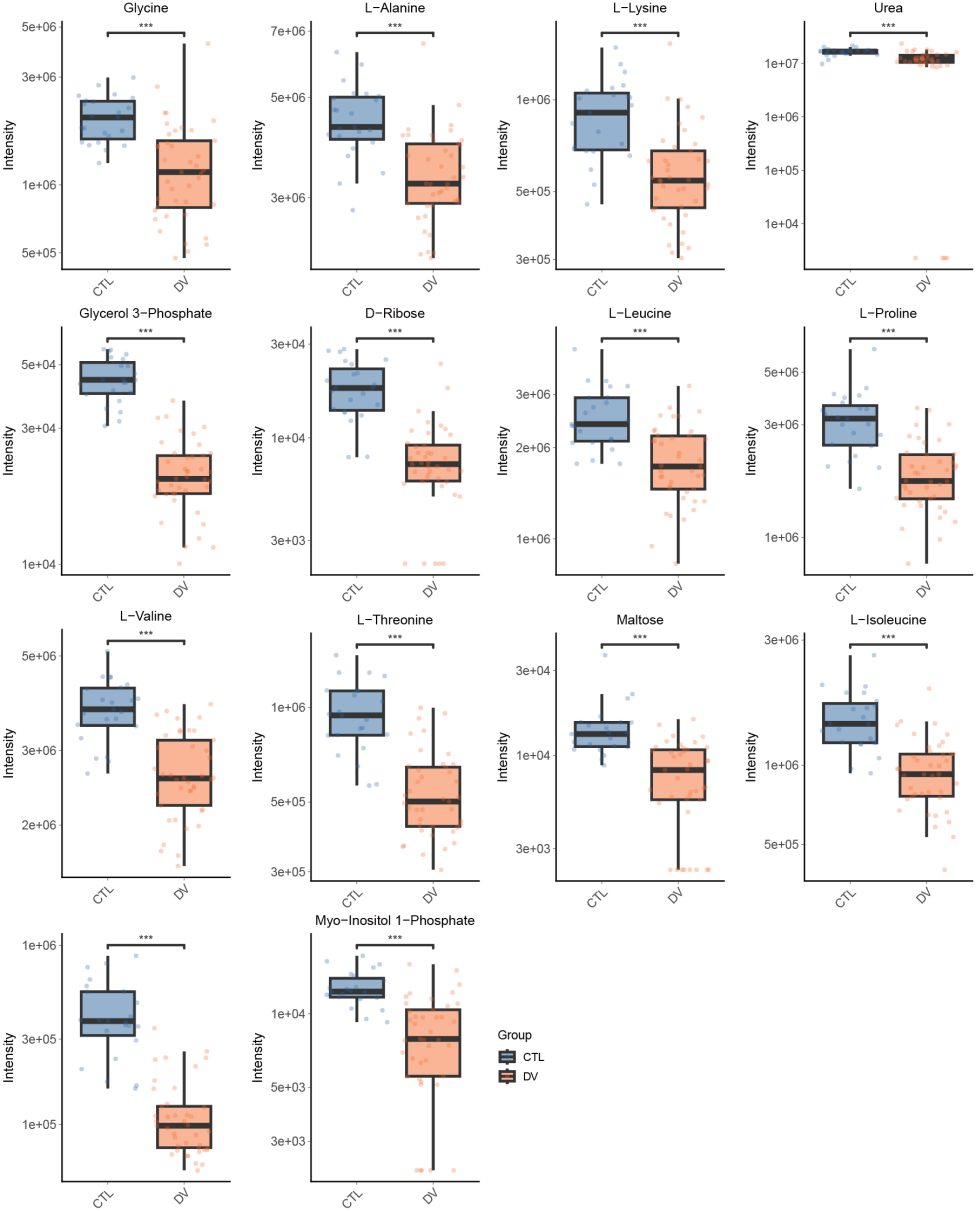
Box plots show the significant changes in metabolite levels of the 14 down-regulated metabolites in ABC transporters. Data were expressed as means ± SE. *** indicates *P* < 0.001. The color key identifies CTL with blue and DV with orange boxes.

We performed pathway enrichment analyses for both up- and down-regulated metabolites (**Figure 5A**). Notably, ferroptosis pathway was enriched in up-regulated metabolites. The ABC transporters pathway was upregulated in some metabolites and downregulated in others. Thus, the results from the metabolite/gene set enrichment analysis for disease (GSEA) were performed to further identify the activation/suppression status of ABC transporters pathway. A graphical representation of the GSEA results in **Figure 5B** will contribute to understanding how the pathways were under the suppression status (NES= -1.47< -1, *P*-value= 0.027<0.05, FDR = 0.199<0.25). Meanwhile, the heat map was used to visualize the trend of expression inhibition of the relevant metabolites (**Figure 5C**). The box plots for each significantly down-regulated metabolite in ABC transporters were constructed to visualize the differences (**Figures 6A–N**).

### 3.5 Identification of differentially expressed genes in ABC transporters pathway

**Figure 7:**
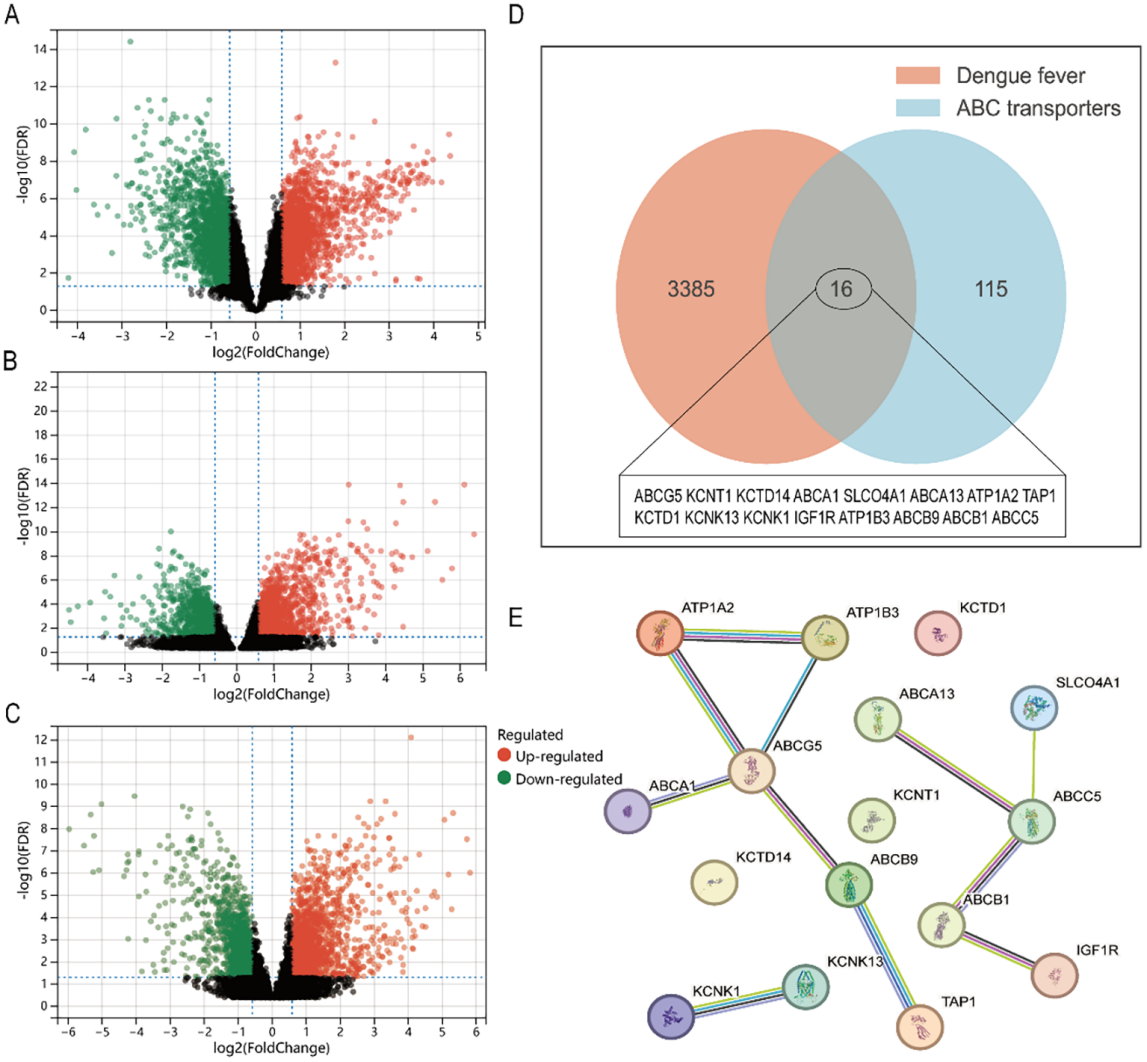
The intersection of dengue fever and ABC transporters is obtained via Venn to focus on the potential core targets. **(A)** Volcano plots for identification of differentially expressed genes in GSE51808, red represents higher gene expression, and green represents lower gene expression. **(B)** Volcano plots for identification of differentially expressed genes in GSE96656. **(C)** Volcano plots for identification of differentially expressed genes in GSE206829. **(D)** 16 potential core targets were identified using Venn diagram. **(E)** protein-protein interaction network showed the relation in the 16 potential core targets.

**Figure 8:**
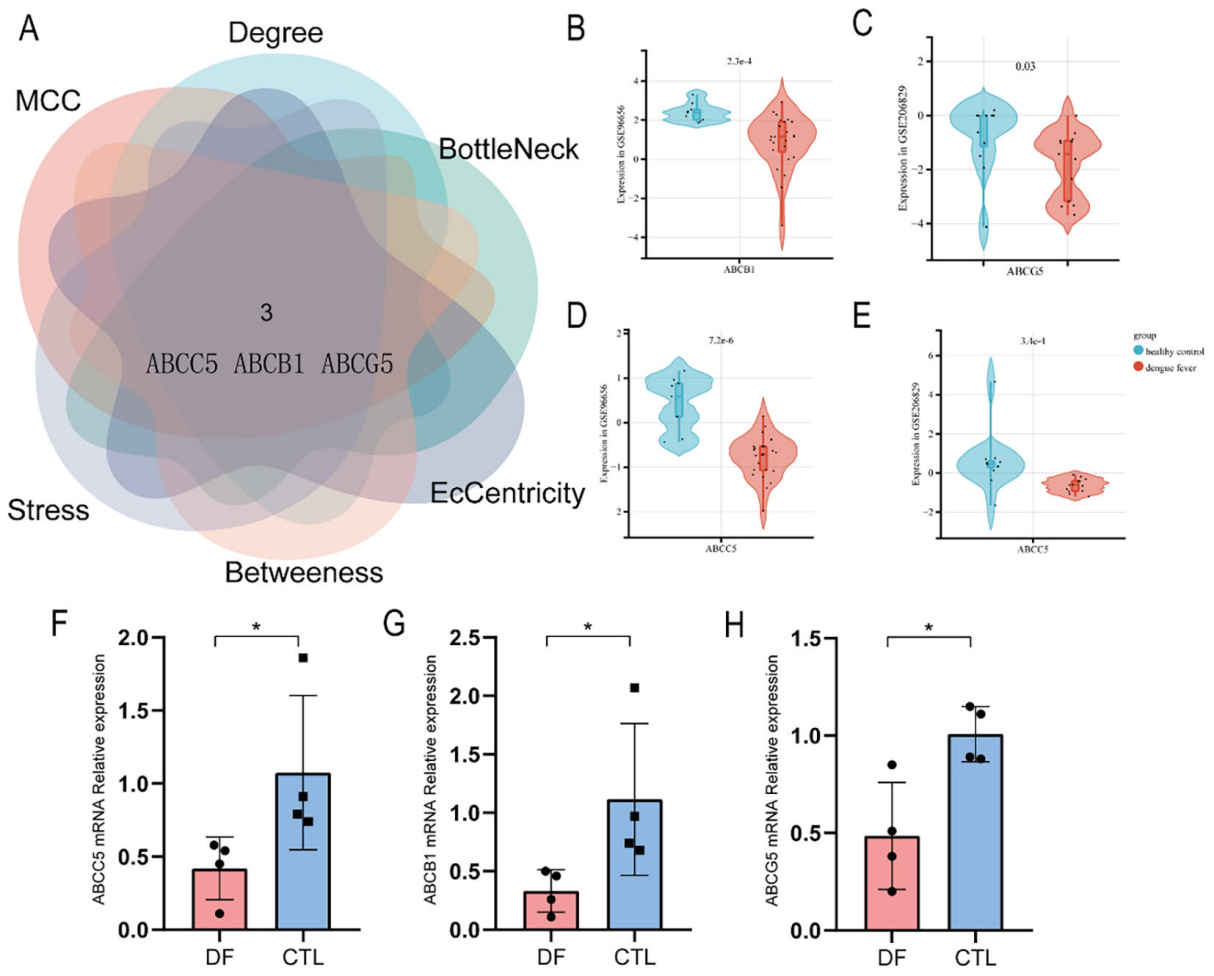
**(A)** Hub genes (ABCC5, ABCB1, and ABCG5) were identified via the different algorithms using the CytoHubba plugin. **(B)** The ABCB1 expressed was lower in dengue fever compared to healthy control in the GSE96656 dataset. **(C)** The ABCG5 expressed was lower in dengue fever compared to healthy control in the GSE206829 dataset. **(D, E)** The ABCC5 expressed was lower in dengue fever compared to healthy control in the GSE96656 and GSE206829 datasets. **(F-H)** The ABCC5, ABCB1, and ABCG5 expressed were lower in dengue fever compared to healthy control in the whole blood by RT-qPCR analyses. * indicates *P* < 0.05.

**Figure 9:**
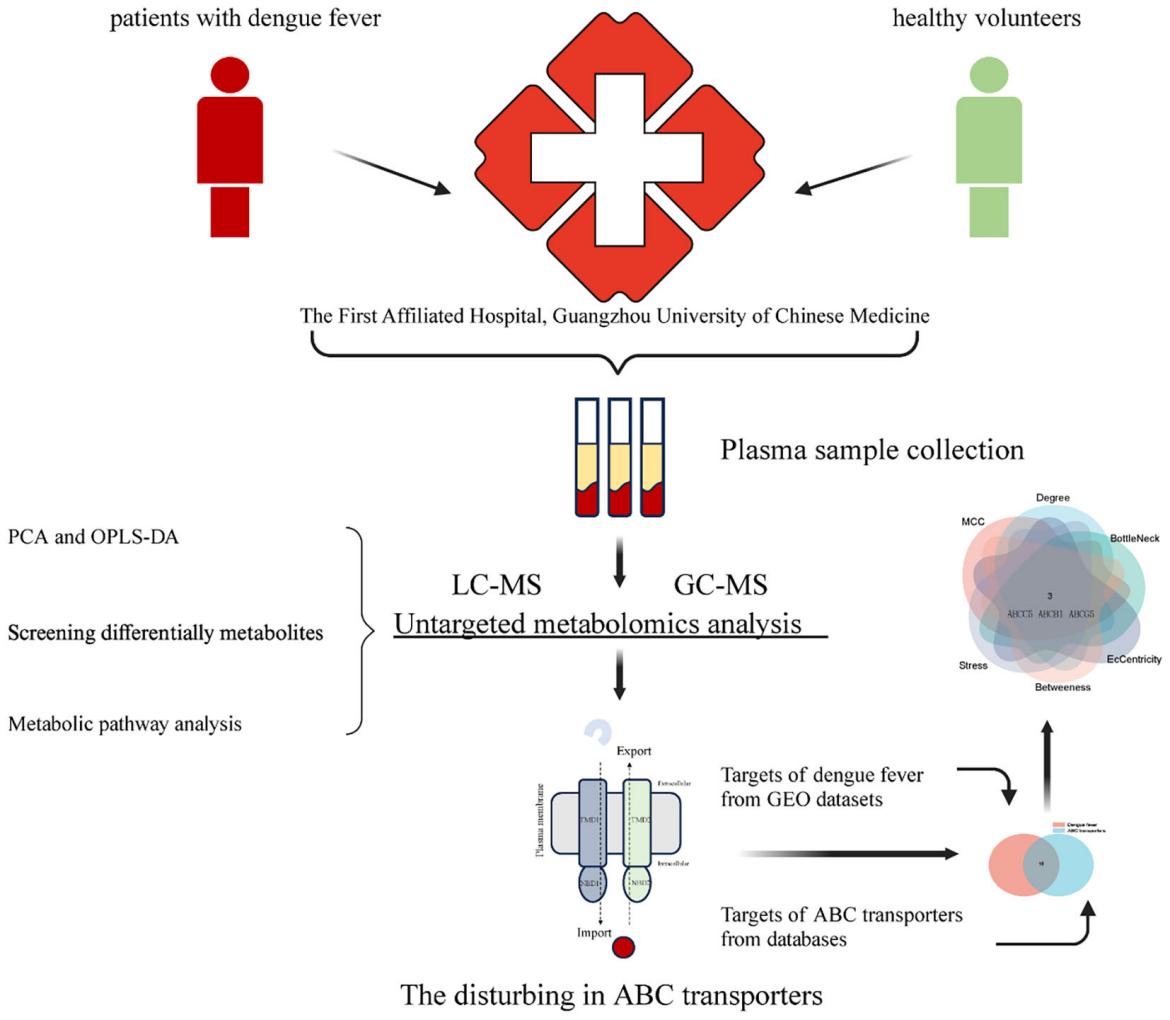
The flowchart of the study.

A total of 3401 genes were differentially expressed in datasets of dengue fever (specific screening conditions are |Log FC| > 1, p-value < 0.05, **Figures 7A-C**). GeneCards and CTDs contained 110 and 44 ABC transporters pathway targets, respectively, for a total of 131 targets included in the final file (**Figure 7D**). Finally, 16 cross-targets of dengue with ABC transporters were obtained. **Figure 7E** showed the correlation network and then, using the CytoHubba plug-in, 3 central genes (ABCC5, ABCB1, and ABCG5) were identified via the different algorithms, and showed a down-regulation trend in the transcriptome data (**Figures 8A–E**). Then the RT-qPCR analyses were conducted to validate the RNA-seq experiments (**Figures 8F–H**). The primer sequences were shown as **Table 4**. At last, a flow chart of the study is shown in **Figure 9**.

**Table 4:** Primer sequence for RT-qPCR.

Gene name	Primer sequence
ABCC5	Forward: GGCTCATCCTGTCCATCGTGTG
	Reverse: TGACCGCAGAATACAGCCATCC
ABCB1	Forward: TTGACAGCTACAGCACGGAAGG
	Reverse: CTTCTTCACCTCCAGGCTCAGTC
ABCG5	Forward: ACGCTGGGCTTACATCCTGAG
	Reverse: GGACGATACCAAGTAGCACAAGAG

## 4 Discussion

Platelet recognition, activation, and aggregation will be promoted after infection with DENV, affecting coagulation (Garcia-Larragoiti et al., 2021). To avoid being disturbed by platelets, the plasma preference is above serum (Hagn et al., 2024). Our research presented a comprehensive metabolomic evaluation for plasma samples from dengue fever compared to healthy controls based on LC-MS and GC–MS analysis. We identified 197 significantly altered metabolites and evaluated the association between the identified metabolites and the metabolic pathways using the KEGG database. Further bioinformatics analyses were performed combined with transcriptomics. Our studies confirm that the ABC transporters, especially 3-target signatures characters of ABCC5, ABCB1, and ABCG5 were perturbed after infection with DENV.

### 4.1 Metabolic spectrum characteristics of patients with dengue fever

Compared with previous studies (Cui et al., 2013), the shared differences in metabolism in dengue patients included 2 up- and 5 down-regulation. Oleic acid, one of the up-regulated metabolites, has proved to be a substance known to increase DENV amplification in natural infection, but not increase the production of dengue virus-like particles (VLPs), that restrict the development of live attenuated vaccines (Ramphan et al., 2017). Docosahexaenoic acid (DHA), another up-regulated metabolic, was positively related to the progression to severe dengue and had the action of regulating inflammatory reactions (Villamor et al., 2018). The shared 5 down-regulation metabolite obtained PC (14:0/0:0), PC (16:1(9Z)/0:0), PC (0:0/16:0), LysoPC (0:0/18:2(9Z,12Z)), PC (18:0/0:0), are all involved in the pathway of phospholipids catabolism. Likewise, the phospholipids showed a decreased and reversible-change trend in DENV-infected humanized mice models (Cui et al., 2017). In the present study, the circulating levels in dengue fever patients of Trans-Cinnamic Acid, L-Acetylcarnitine, SM(d17:1/17:0), 1,2,4,5-Cyclohexanetetrol, 5-(Hydroxymethyl) Pyrrolidin-2-One, 1,2,3,4-Tetrahydro-6-Propanoylpyridine, 2-C-Methyl-D-Erythritol-4-Phosphate, Physalolactone, S-Japonin, and 9-Tridecynoic Acid were higher than those in the control group, and with a high AUC value as well, may function as diagnosis biomarkers to distinguish patients infected with DENV.

### 4.2 The significantly enriched pathways in differential metabolites

The previous study (Cui et al., 2013) revealed that major perturbed metabolic pathways included fatty acid biosynthesis and β-oxidation, phospholipid catabolism, steroid hormone pathway, etc. Relative to the previous research, the Biosynthesis of unsaturated fatty acids was identified as one of the enrichment metabolic pathways in this study. Actually, there was a relative study to highlight both saturated fatty acids and unsaturated fatty acids showed a decreasing trend in the dengue fever patients versus healthy volunteers by GC/MS (Khedr et al., 2015). A previous study had reported that stearoyl-CoA desaturase-1 (SCD1) inhibitor showed potent suppression of DENV replication in a dose-dependent manner, however, exogenous supplementation of unsaturated fatty acids resulted in the reversal of the effect of SCD1, suggesting that biosynthesis of fatty acids contributes to viral replication capacity (Hishiki et al., 2019).

In addition, the following metabolic pathway is enriched as follows: Protein digestion and absorption, aminoacyl-tRNA biosynthesis, Mineral absorption, and D-Amino acid metabolism. The pathway of Protein digestion and absorption was found to be suppressed upon downregulation changes in metabolites with DENV infection. Aminoacyl-tRNAs are the essential substrates for translation depending on the catalyzed reaction of synthetase, which is an essential component involved in protein synthesis and may be potent drug targets to anti-viral (Chakraborti et al., 2021). Furthermore, the inhibition of D-Amino acid metabolism was observed in the present study. Furthermore, the inhibition of D-Amino acid metabolism was observed in the present study, there are no reports about these metabolite features in dengue patients. Research has shown that the level of D-amino acid was dynamically changed in COVID-19 patients, low in the severe and increasing before the recovery (Kimura-Ohba et al., 2023).

### 4.3 The disturbance in ABC transporters is a characteristic change

ABC transporters, consisting of a ubiquitous superfamily of integral membrane proteins, characteristic of two nucleotide-binding domains (NBDs) and two transmembrane domains (TMDs), have the function of the ATP-powered translocation of many substrates across membranes (Rees et al., 2009). ABC transporters are relevant to the pathological process in broad human diseases and thus may be promising targets for drug discovery (Moore et al., 2023). The ABC transporters may play a key role in arboviral infection and immunity, previous research had reported that most transporters of the ABC subfamily were downregulated via the transcriptomics data (Kumar et al., 2021). Likewise, ABC transporters were altered in the mice’s urine with DENV infection (Zheng and Wang, 2022). However, those metabolic items research of ABC transporters have not been sufficiently differentiated in human dengue patients. We provided the first *in vivo* evidence that dengue virus infection disrupts the function of ABC transporters in human metabolism. This present study revealed that the ABC transporters pathway was under suppression status. We intended to describe the major disturbance metabolism pathway of ABC transporters in patients with dengue fever, and thus be viewed as the foundation, with more detailed and in-depth studies on the impact to follow. ABC transporters have the function of mediating the uptake of various nutrients, our findings indicated that Protein digestion and absorption, aminoacyl-tRNA biosynthesis, Mineral absorption, and D-Amino acid metabolism were all affected while DENV-infected. From this, the disturbance of ABC transporters is not an isolated event, but rather a key mechanism that causes or exacerbates abnormal distribution of critical intracellular metabolites, thereby impacting viral replication or immunopathology.

### 4.4 ABCC5, ABCB1, and ABCG5 are identified as hub differentially expressed targets

ABCC5, ABCB1, and ABCG5 are distributed in distinct families of human ABC transporters and have the function of encoding for membrane transporters, and the dysfunctions are involved in many disease pathways (Alam and Locher, 2023). ABCC5 was proved as a general glutamate conjugate and analog transporter, with the effect of limiting brain levels of endogenous metabolites, drugs, and toxins (Jansen et al., 2015). ABCB1, member 1 in the ATP-binding cassette subfamily B, protects vital organs from outside chemicals and expels medications from malignant cells. The inhibitor molecular to ABCB1 would likely be prescribed as a potential anti-cancer drug (Patel et al., 2024). Moreover, ABCB1 had set complex and diverse regulatory mechanisms in oxidative stress (OS), which mainly exerts a protective effect by preventing the penetration and removing the production of OS, however, it also decreased under the instances with severe OS (Shchulkin et al., 2024). ABCG5 transporters can keep xenosterols away from accumulating in the human body (Patel et al., 2018), the gene variation of ABCG5 would lead to balance disruption and subsequently cause bleeding abnormality and macrothrombocytopenia (Kanaji et al., 2013). Interestingly, thrombocytopenia is a common factor that increases the risk of bleeding in patients with dengue fever, but platelet transfusions are unlikely to improve the prognosis (Archuleta et al., 2020). Based on the analyses above, we propose the potential mechanism that DENV may interfere with the function of the ABC transporter pathway in the host, specifically targeting ABCC5, ABCB1, and ABCG5, and thus enhance disease.

## 5 Conclusion

The current study aims to explore the metabolome change in DENV-infected patients compared to healthy people, and the hub gene being disturbed in the ABC transporters. We identified 61 up-regulated and 136 down-regulated metabolites. The top 10 up-regulated metabolites have some potential to indicate diagnostic biomarkers in dengue fever. Many metabolic pathways which involve Protein digestion and absorption, aminoacyl-tRNA biosynthesis, Mineral absorption, and D-Amino acid metabolism, were all affected. The disturbance of ABC transporters was identified as the characteristics of metabolic pathways in patients with dengue fever. ABCC5, ABCB1, and ABCG5 were identified as hub differentially expressed targets via transcriptomics. These findings shed new light on the mechanism underlying metabolomics of dengue fever, particularly the disturbance in ABC transporters. Further investigation of the effect of ABC transporters on dengue fever would need to be done.

## 6 Limitation

Our study is based on data mining integration of metabolomics and transcriptomics, reveals the distinctive molecular signatures of patients infected with DENV, and will stimulate further structural and functional studies of ABC transporters. We recommend that molecular and experimental validation of the function of the ABC transporter is crucial for further exploration. Second, the metabolite profiling in this study was conducted only using the strategy of untargeted metabolomics, the targeted metabolomics assay would be considered for quantification analysis. Thirdly, does the addition of the down-regulated metabolites have an improved response following DENV-infected? The study will be useful as a reference for our future research topic. Fourth, the severity of infection, viral serotype, and days post-infection may all influence the metabolic profile. However, due to the small sample sizes within each subgroup, we did not conduct further analysis of these factors based on subgroup information. Furthermore, this study identified several potential or candidate biomarkers, future research will collect more samples for validation.

## Data Availability

All data are provided in article form and presented in tables, figures, and supplementary files. The raw data included in the present study are available from the corresponding authors (SFZ) upon request. The metabolomics data have been deposited in the Open Archive for Miscellaneous Data (OMIX, https://ngdc.cncb.ac.cn/omix/) with the identifier OMIX013705.
